# Altered microRNA profiles during early colon adenoma progression in a porcine model of familial adenomatous polyposis

**DOI:** 10.18632/oncotarget.21774

**Published:** 2017-10-10

**Authors:** Monika Stachowiak, Tatiana Flisikowska, Stefan Bauersachs, Carolin Perleberg, Hubert Pausch, Marek Switonski, Alexander Kind, Dieter Saur, Angelika Schnieke, Krzysztof Flisikowski

**Affiliations:** ^1^ Department of Genetics and Animal Breeding, Poznan University of Life Sciences, 60-637 Poznan, Poland; ^2^ Chair of Livestock Biotechnology, Technische Universität München, 85354 Freising, Germany; ^3^ Institute of Agricultural Sciences, Animal Physiology, ETH Zurich, CH-8092 Zurich, Switzerland; ^4^ Current address: University of Zurich, Clinic for Animal Reproduction Medicine, Genetics and Functional Genomics Group, CH-8092 Zurich, Switzerland; ^5^ Institute of Agricultural Sciences, Animal Genomics, ETH Zurich, CH-8092 Zurich, Switzerland; ^6^ Klinikum Rechts der Isar II, Technische Universität München, 81675 Munich, Germany

**Keywords:** colorectal cancer, dysplasia, isomiR, microRNA, pig model

## Abstract

MicroRNAs are dysregulated in various cancers including colorectal cancer, and are potential useful biomarkers of disease development. We used next generation sequencing to investigate miRNA expression profiles in low- and high-grade intraepithelial dysplastic polyps from pigs carrying a mutation in the adenomatous polyposis coli tumour suppressor (*APC^1311^*, orthologous to human *APC^1309^*) that model an inherited predisposition to colorectal cancer, familial adenomatous polyposis. We identified several miRNAs and their isomiRs significantly (*P* < 0.05) differentially expressed between low and high-grade intraepithelial dysplastic polyps. Of these, ssc-let-7e, ssc-miR-98, ssc-miR-146a-5p, ssc-miR-146b, ssc-miR-183 and ssc-miR-196a were expressed at higher level and ssc-miR-126-3p at lower level in high-grade intraepithelial dysplastic polyps. Functional miRNA target analysis revealed significant (*P* < 0.001) over-representation of cancer-related pathways, including ‘microRNAs in cancer’, ‘proteoglycans in cancer’, ’pathways in cancer’ and ‘colorectal cancer’. This is the first study to reveal miRNAs associated with premalignant transformation of colon polyps.

## INTRODUCTION

MicroRNAs are short (~22nt) non-coding RNAs that are widely implicated in translational repression and degradation of target mRNAs, and estimated to down-regulate up to 60% of genes [1]. They are emerging as predictive and prognostic biomarkers and as therapeutic targets in a wide range of human diseases, including cancer [2]. Cellular and circulating miRNAs have been linked to colorectal cancer (CRC), a common and severe form of cancer that often arises from adenomas (polyps) in the colon and rectum [3, 4].

Colorectal cancer is the third most commonly diagnosed cancer in males, second in females, and estimated to cause more than 600,000 deaths per year worldwide [5]. Lifestyle risk factors include smoking, physical inactivity, overweight, consumption of red and processed meat, and alcohol; but there is also a strong genetic component to susceptibility [6, 7]. Development of strategies to prevent, diagnose and treat CRC will be aided by animals that model the disease and predict efficacy in humans.

The pig is increasingly recognised as a useful model species in translational medicine. Genetically modified pigs carrying oncogenic mutations similar to lesions responsible for human cancers could provide a valuable resource for preclinical studies [8]. We previously generated pigs carrying a germ line *APC^1311^* mutation, orthologous to human *APC^1309^*, to model familial adenomatous polyposis (FAP) [9]. FAP patients develop adenomatous polyps in the colon and rectum and are strongly predisposed to colorectal cancer [10, 11]. *APC^1311^* pigs develop dysplastic adenomas closely resembling early stage human FAP, and the progression of adenomatous polyps shows typical epithelial features of the adenoma-carcinoma sequence including aberrant crypt foci, adenomatous polyps with low (LG-IEN) and high-grade intraepithelial (HG-IEN) dysplasia and carcinoma *in situ* [9]. More recently, we found that similar sets of protein-coding genes were involved in premalignant transformation in *APC^1311^* pigs as in human CRC pathogenesis [12]. The aim of this study was to identify miRNAs associated with progression from low- to high-grade intraepithelial dysplasia.

## RESULTS AND DISCUSSION

### Identification of differentially expressed miRNAs

**Figure 1:**
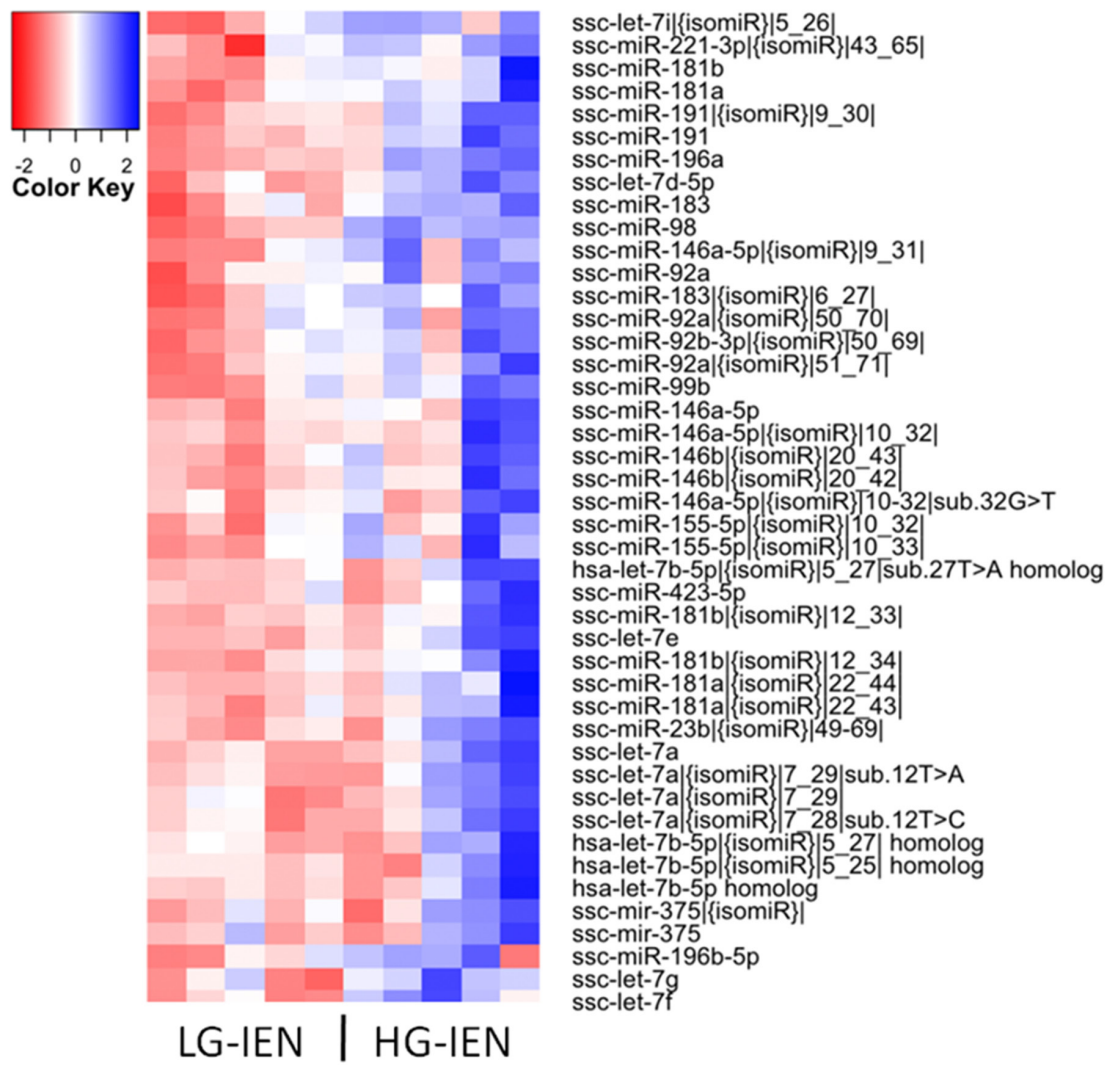
Heatmap showing 44 differentially expressed mature miRNAs and isomiRs in low-grade intra-epithelial dysplasia (LG-IEN) and high-grade intra-epithelial dysplasia (HG-IEN) samples based on the normalised Log2-transformed fold change values and the *P*-value

To identify miRNAs associated with early stage adenoma progression, we performed miRNA profiling of normal colonic mucosa (NM), LG-IEN and HG-IEN samples using next generation sequencing. Cluster analysis revealed the most distinct expression patterns between LG-IEN and HG-IEN groups (Figure 1; Supplementary Figure 1). In total, more than 1200 small RNA sequences were analysed. Of these, only six miRNAs, including two isomiRs, showed differential expression (three up-, and three down-regulation) (*P* < 0.05) at the earliest stage of adenoma progression, i.e. between NM and LG-IEN samples (Supplementary Table 2). The expression level of another 44 miRNAs, including 26 isomiRs of known mature miRNAs, increased during progression from LG-IEN to HG-IEN (Supplementary Table 2). Between NM and HG-IEN samples we identified 41 differentially expressed miRNAs (21 up-regulated and 20 down-regulated), including 31 isomiRs of known miRNAs (Supplementary Table 2).

Most (64%) differentially expressed miRNAs identified in our study were isomiRs (Supplementary Table 2). These are variants of mature, canonical miRNAs that differ in sequence at the 3’ or 5’ ends, or harbour internal substitutions [13]. Most (77%) variations we identified involved the 3’ end, which is a common observation [14]. IsomiRs have been implicated in various biological pathways on the basis that their distribution differs consistently between cell types. For example, their expression profile differs between normal and cancer tissue and they may be used to discriminate various types of cancer [15]. Canonical miRNAs and their isomiRs are also known to act in co-ordination to target common molecular pathways [16]. We therefore performed a further analysis using a common identifier of the canonical miRNAs.

### miRNA targets and pathway analysis

To determine possible biological roles of differentially expressed microRNAs in polyp development, we performed functional enrichment analysis based on their target mRNAs. Tools to predict porcine miRNA targets are not yet available, but porcine and human mature microRNA orthologues are highly conserved [17] enabling the use of human databases. MicroRNAs differentially expressed between NM and LG-IEN samples targeted 7743 genes. A total of 8339 transcripts were targets of miRNAs up-regulated between LG-IEN and HG-IEN adenoma. Finally, 9794 genes were regulated by miRNAs differentially expressed between NM and HG-IEN samples (Supplementary Table 3). The pathways most significantly enriched (*P* ≤ 3.75 × 10^-11^) during early adenoma progression and targeted by the highest number of miRNAs were related to ‘microRNAs in cancer’, ‘proteoglycans in cancer’, ‘hepatitis B’ and ‘cell cycle’ (Supplementary Table 4). Many other cancer-related pathways were also significantly affected (*P* < 0.001), including ‘viral carcinogenesis’, ’pathways in cancer’, ‘colorectal cancer’, ‘p53 signalling pathway’, and ‘transcriptional misregulation in cancer’. Interestingly, the involvement of multiple pathways related to cancer was observed even at the earliest stage of premalignant transformation i.e. during progression to LG-IEN, and of these the ‘microRNAs in cancer’ pathway was most significantly affected (*P* = 1.83 × 10^-63^) (Supplementary Table 4).

### Validation of candidate miRNAs

**Figure 2:**
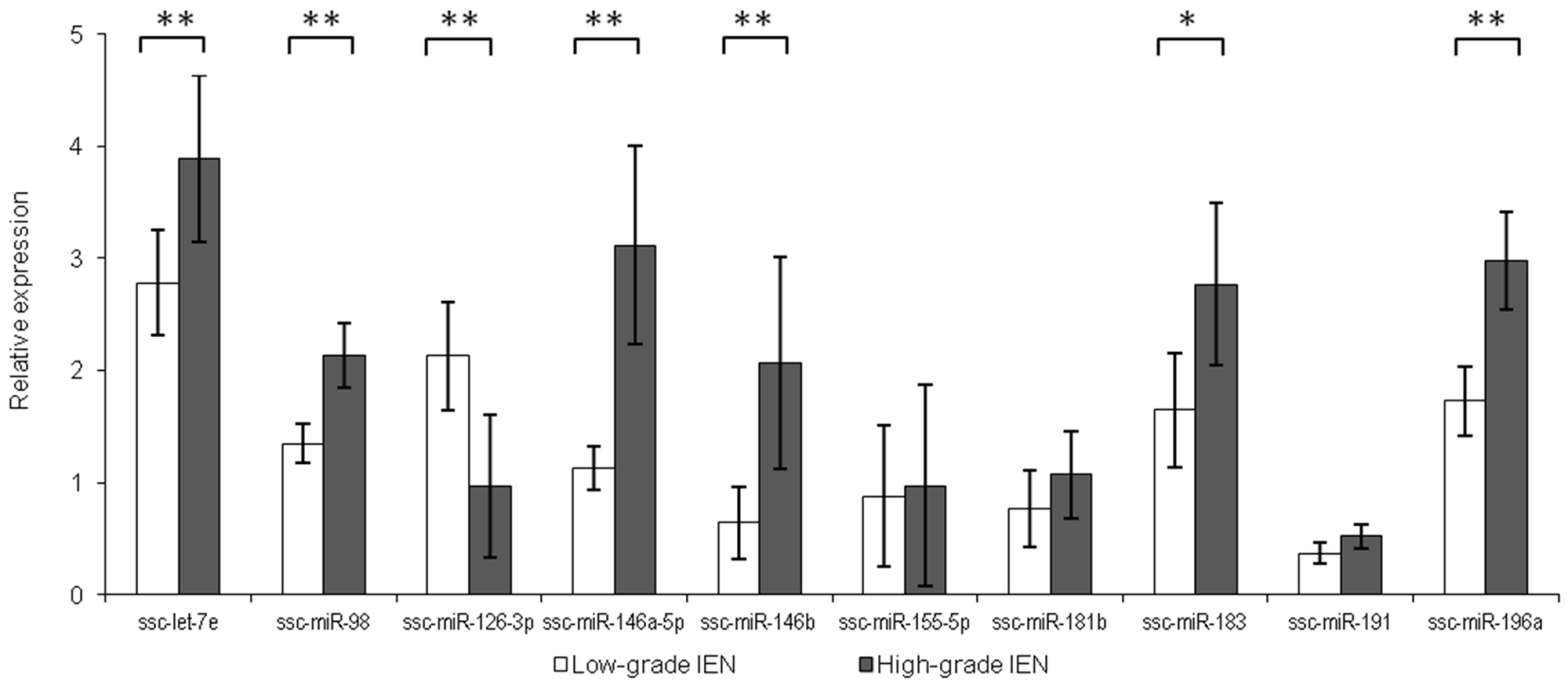
Relative miRNA expression in low-grade intra-epithelial dysplasia (LG-IEN) and high-grade intra-epithelial dysplasia (HG-IEN) samples Values are means ± SD presented in relation to normal mucosa. *P < 0.05, ^**^P < 0.01.

Ten differentially expressed miRNAs were validated by qRT-PCR: ssc-let-7e, ssc-miR-98, ssc-miR-126-3p, ssc-miR-146a-5p, ssc-miR-146b, ssc-miR-155-5p, ssc-miR-181b, ssc-miR-183, ssc-miR-191 and ssc-miR-196a. These were selected on the basis of ranking in the list of differentially expressed miRNAs and predicted functional relevance for CRC progression. In the more advanced HG-IEN samples, the levels of ssc-let-7e, ssc-miR-98, ssc-miR-146a-5p, ssc-miR-146b, ssc-miR-183 and ssc-miR-196a were significantly elevated compared to LG-IEN samples. In contrast, ssc-miR-126-3p was significantly reduced (Figure 2). Differences in ssc-miR-155-5p, ssc-miR-181b and ssc-miR-191 levels fell below statistical significance, but were still consistent with data from small RNA sequencing (Figure 2).

MicroRNAs can act to either promote or suppress tumorigenesis by regulating the expression of genes involved in angiogenesis, apoptosis, cell cycle control and differentiation. Members of the let-7 family are broadly considered to be tumour suppressors, downregulating several oncogenes including *KRAS* [18]. However, let-7e upregulation has been observed in human CRC tumours and associated with poor prognosis [19]. Interestingly, and consistent with the human data, we also found elevated levels of let-7e in HG-IEN. The microRNA miR-98 has been found to play a suppressive role in various human malignancies, but its role in CRC development is unclear. It has been recently reported that miR-98 was down-regulated in colon cancer tissues compared to adjacent mucosa [20], but we found it significantly increased with precancerous adenoma development. We also found that ssc-miR-146a-5p and ssc-miR-146b were expressed at higher level in HG-IEN samples, which accords with findings in humans that these are expressed at higher levels in colon tumours than adjacent normal tissue, and are involved in CRC invasion [21, 22]. In our study we detected upregulation of ssc-miR-183 in HG-IEN; similarly elevated levels of mir-183 is associated with human colorectal carcinogenesis [23, 24]. Finally, we found higher expression of ssc-miR-196a in HG-IEN; human miR-196a contributes to cell growth promotion, migration and invasion of colorectal cancer cells [25–27]. We also found that ssc-miR-126 was expressed significantly less in HG- than in LG-IEN. Downregulation of miR-126 is observed during colon carcinogenesis in humans, and is associated with cancer cell proliferation, migration and invasion [28, 29].

Interestingly, a recently published comparison of human colon carcinomas with adjacent normal tissues showed a global up-regulation and a different set of miRNAs associated with carcinogenesis [30]. However, none of the eight miRNAs indicated in that study as potential diagnostic biomarkers and targets for CRC treatment showed differential expression in our samples. We also found only low and unchanged expression of the microRNAs, ssc-miR-155-5p, ssc-miR-181b and ssc-miR-191, even though they have previously implicated in CRC development in humans [31–33]. It is likely that these differences reflect the earlier premalignant stage of disease development that we analysed, compared to the human data obtained from biopsies of later stages of CRC.

A study by Oberg et al. [34] of miRNAs expressed during the development of human sporadic and hereditary non-polyposis colorectal cancer (HNPCC) revealed more than 30 differentially expressed species. Their comparison of normal mucosa with adenoma showed involvement of the same miRNAs and a similar magnitude of difference as comparison of normal mucosa with later stage carcinoma [34], leading them to suggest that changes in miRNAs occur at an early stage persist during later stages of tumorigenesis in both proficient and defective DNA mismatch repair tumours. While our study revealed a few similarities with their study, several differentially expressed miRNAs such as miR-135a-1, miR-135a-2, miR135b showed no change in the FAP pigs. Where this discrepancy arises is unclear, but could be due to differences between HNPCC and FAP. As such, direct comparison of the porcine FAP model with early stage human adenomas would be informative.

We recently reported [12] that the use of whole biopsy samples of adenomas can introduce variability and reduce the sensitivity of differential transcriptome analysis as a result of cell heterogeneity, especially in the more advanced polyps. Laser-guided microdissection and collection of defined regions of adenoma avoided the obscuring effect of events in other tissue compartments and provided a more defined and sensitive analysis of adenoma progression. This effect is also likely to apply to the data presented here (Figure 1 and Supplementary Figure 1B). Although technically difficult at present, future analysis of microdissected adenoma would reduce such variability and probably reveal other miRNAs relevant to early adenoma progression.

In summary, we have detected several miRNAs (ssc-let-7e, ssc-miR-98, ssc-miR-126-3p, ssc-miR-146a-5p, ssc-miR-146b, ssc-miR-183 and ssc-miR-196a) associated with early-stage colorectal neoplasia in *APC^1311^* pigs. Aberrant expression of these miRNAs can be considered as a molecular signature of precancerous progression in colorectal polyps, and they represent potential targets for early CRC therapy.

## MATERIALS AND METHODS

### Ethics statement

All experimental procedures were conducted in accordance with the ethical standards, approved by the Government of Upper Bavaria (permit number 2 55.2-1-54-2532-6-13) and performed according to the German Animal Welfare Act and European Union Normative for Care and Use of Experimental Animals (EU Directive 2010/63/EU).

### Animals and sample collection

*APC^1311/+^* pigs were fed with normal pig diet. Polyp and normal mucosa samples were collected during colonoscopy examinations carried out approximately every 6 months, starting at 3 months old. Tissue samples were snap frozen in liquid nitrogen and stored at -80°C for subsequent analyses. For histopathology assessment, specimens were fixed in 4% buffered paraformaldehyde, embedded in paraffin, sectioned (3 μm) and stained with haematoxilin and eosin (H&E). Polyps were classified as LG-IEN (low grade intra epithelial dysplasia) or HG-IEN (high grade intra epithelial dysplasia) according to the AJCC TNM staging system [35].

### Library preparation and NGS small RNA sequencing

Total RNA was purified using a Zymo Direct-zol RNA MiniPrep kit (Zymo Research). The quality and quantity of RNA samples was measured using an Agilent RNA 6000 Nano kit (Agilent) on a 2100 Bioanalyzer (Agilent) and a Nanodrop 2000 spectrophotomoter (Thermo Scientific). Libraries for small RNA sequencing were prepared (normal colon mucosa, NM, n=2; LG-IEN, n=5; HG-IEN, n=5) using 1 μg total RNA with TruSeq Small RNA Library Prep Kit (Illumina) according to the manufacturer's instructions. Twelve samples were pooled and after validation (Agilent 2100 Bioanalyzer) run on MiSeq system (Illumina) using MiSeq Reagent Kits v2(Illumina) at 1×50 bp read configuration to generate FASTQ files.

### Bioinformatic analysis of sequencing data

Analysis of Illumina sequence data was performed on a locally installed Galaxy server [36]. The first step was to detect and remove adapter sequences with the tool FastqMcf (Galaxy Version 1.0). The quality of Fastq files was checked before and after adapter clipping with FastQC (v0.11.2) and multiqc (Galaxy Version 0.6) to monitor performance of the processing step [37]. Since each resulting sequence should correspond to a small ncRNA, the data analysis strategy was to generate a count table for all unique sequences obtained as a basis for subsequent statistical analysis and to annotate and identify differentially expressed sequences. A series of standard Galaxy tools and additional converting tools from the ToolShed (https://toolshed.g2.bx.psu.edu/) were used. Initially, each adapter-clipped FastQ file was collapsed into the sequence and the number of appearances (using the tool “Collapse” - FastxTools http://hannonlab.cshl.edu/fastx_toolkit/) ranked by the counts. The resulting FASTA files contained the counts and rank of the corresponding sequence in each header. FASTA files were then converted into Galaxy data file type with the tool “FASTA-to-Tabular” (Version 1.1.0). The final count table containing only the sequences and the number of reads per sample was derived using an in-house tool named “Join datasets by identifier column” (https://toolshed.g2.bx.psu.edu/) and Galaxy (Version 1.0.2). This count table was filtered to remove sequences with negligible read counts (most likely sequencing artifacts), using 33 counts per million (CPM) per sample in at least two samples. This resulted in 3214 sequences passing the filter. These sequences were then compared to all transcripts of *Sus scrofa* including non-coding RNAs and human and bovine sequences with NCBI BLAST+ [38]. The collection of BLAST databases contained precursor and mature microRNAs sequences from miRBase, sequences from NCBI and Ensembl, mostly non-coding RNAs but also protein-coding transcripts, as well as tRNA and piRNA cluster sequences retrieved from NCBI. Porcine tRNA sequences were extracted from the genome sequence (Sus Scrofa 10.2) using the GFF3 annotation file available at NCBI. All BLAST results were filtered and joined by removing duplicated hits. Sequences ranging from 19 to 24 nt assigned to miRNAs were used for analysis of differential expression with EdgeR [39]. IsomiRs were annotated based on the mature and step-loop miRNAs sequences deposited in miRBase v. 21, applying nomenclature proposed by Cloonan *et al.* [16].

### MicroRNA target prediction and functional enrichment

MicroRNA target genes and KEGG (Kyoto Encyclopedia of Genes and Genomes) pathways influenced by differentially expressed miRNAs were predicted using DIANA-miRPath v3.0 software [40, 41]. Experimentally validated human miRNA:mRNA interactions available in TarBase v.7.0 were applied. The resulting significance levels were adjusted for false discovery rate to take account of multiple testing.

### Quantitative RT-PCR

To validate the small RNA sequencing results, forty RNA samples (NM, n=12; LG-IEN, n=24; HG-IEN, n=24) were reverse transcribed using a miScript II RT kit (Qiagen). Quantification of 10 differentially expressed miRNAs was performed in triplicate by real-time PCR on an ABI 7500 thermocycler (Applied Biosystems) using a miScript SYBR Green PCR kit (Qiagen) according to the manufacturer's recommendations. Primer specificity was verified by melting curve analysis. The sequence of custom-designed (www.qiagen.com/miDesign) PCR primers is shown in Supplementary Table 1. Relative expression level was calculated by the ΔΔCt method and miR-7-5p was used as a reference miRNA to normalise the results. The expression of miRNAs in LG-IEN and HG-IEN were compared to normal mucosa. Students t test was used to compare LG-IEN and HG-IEN samples.

## SUPPLEMENTARY MATERIALS FIGURES AND TABLES








