# Supplementary material for: Altered microRNA profiles during early colon adenoma progression in a porcine model of familial adenomatous polyposis
Source: Oncotarget. 2017 Oct 10;8(56):96154–60. doi: 10.18632/oncotarget.21774 (PMC5707088; doi:10.18632/oncotarget.21774)
Supplement: Supplementary file 1 [file oncotarget-08-96154-s001.pdf]

Altered microRNA profiles during early colon adenoma progression in a porcine model of familial adenomatous polyposis

SUPPLEMENTARY MATERIALS

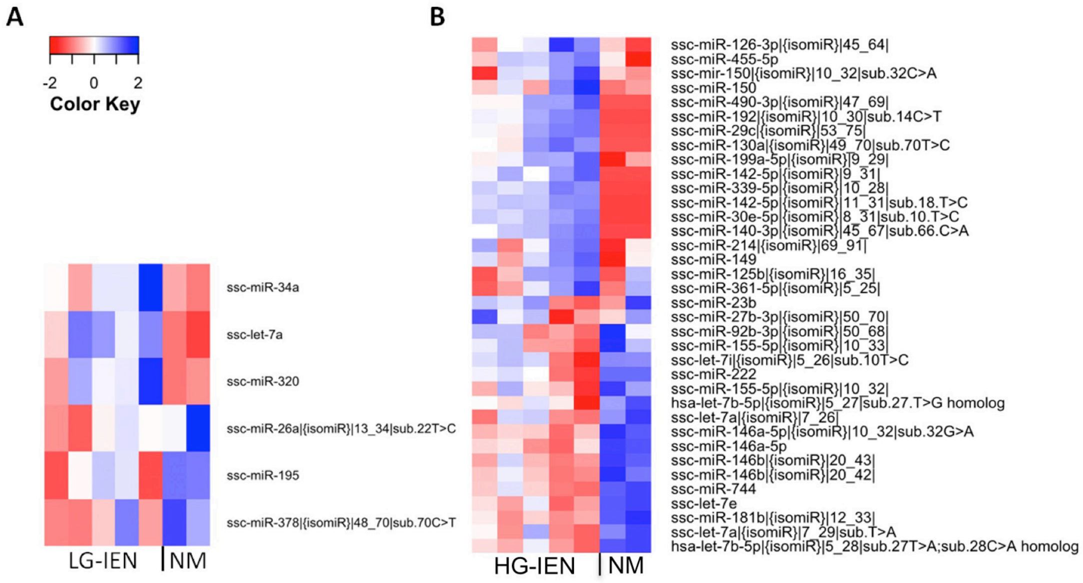

Supplementary Figure 1: Heatmaps showing differentially expressed mature miRNAs and isomiRs based on the normalised Log2-transformed fold change values and the P-value between: **(A)** low-grade IEN (LG-IEN) and normal mucosa (NM); **(B)** high-grade IEN (HG-IEN) and normal mucosa (NM).

Supplementary Table 1: Primers used for validation of selected miRNA expression by real-time PCR analysis

miRNA	Forward Primer (5'→3')[1]
ssc-let-7e	TGAGGTAGGAGGTTGTAT
ssc-miR-98	GGTAGTAAGTTGTATTGTT
ssc-miR-126-3p	TCGTACCGTGAGTAATAA
ssc-miR-146a-5p	TGAGAACTGAATTCCATGG
ssc-miR-146b	AACTGAATTCCATGGGTTA
ssc-miR-155-5p	TTAATGCTAATTGTGATAGG
ssc-miR-181b	AACATTCATTGCTGTCGGTG
ssc-miR-183	GGCACTGGTAGAATTCACT
ssc-miR-191	ACGGAATCCCAAAAGCAGC
ssc-miR-196a	TAGGTAGTTTCATGTTGTT

MiScript universal primer (Qiagen) was used as reverse primer.

Supplementary Table 2: Differentially expressed microRNAs and isomiRs between normal mucosa, low-grade IEN and high-grade IEN samples.

See Supplementary File 1

Supplementary Table 3: Target genes for differentially expressed microRNAs between normal mucosa, low-grade IEN and high-grade IEN samples.

See Supplementary File 2

Supplementary Table 4: KEGG pathways significantly enriched by differentially expressed microRNAs between normal mucosa, low-grade IEN and high-grade IEN samples.

See Supplementary File 3
